# Tuning Tribological Performance of Layered Zirconium Phosphate Nanoplatelets in Oil by Surface and Interlayer Modifications

**DOI:** 10.1186/s11671-017-2315-2

**Published:** 2017-09-20

**Authors:** Xiao Han, Huaisong Yong, Dazhi Sun

**Affiliations:** Department of Materials Science and Engineering and Shenzhen Key Laboratory of Nanoimprint Technology, Southern University of Science and Technology, Shenzhen, 518055 China

**Keywords:** Layered nanoplatelets, Nanolubricants, Zirconium phosphate, Surface modification, Intercalation

## Abstract

Two-dimensional (2D) inorganic layered nanoplatelets exhibit superior lubricating properties in both solid states and oil dispersions. In this paper, we have systematically investigated the effects of surface and interlayer modifications on the tribological performance of layered α-zirconium phosphate (ZrP) nanoplatelets in mineral oil. The pristine layered ZrP nanoplatelets were first reacted with silanes of different alkyl chains to achieve outer surface modifications, followed by intercalation with different alkyl amines to alter the interlayer spacing. Friction and anti-wear studies on ZrP nanoplatelets with various modifications in mineral oils suggest that a longer alkyl chain on the outer surfaces along with a small increase in interlayer spacing would lead to a better tribological behavior especially under a relatively heavy load condition. Our results illustrate the ability of tuning the tribological properties of 2D layered nanoplatelets in oils by varying their surface and interlayer functionalities and would be helpful for understanding the underlying tribological mechanisms of nanolubricating oils containing 2D layered nanoplatelets.

**Electronic supplementary material:**

The online version of this article (10.1186/s11671-017-2315-2) contains supplementary material, which is available to authorized users.

**Graphical abstract:**
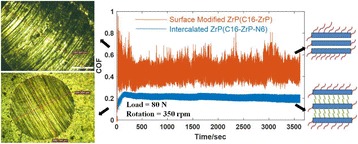
ᅟ

## Background

Lubricating oils containing inorganic nanoparticles, also called nanolubricating oils, have drawn extensive attentions in both scientific and industrial communities due to their superior friction and anti-wear properties as compared to the conventional lubricating oils with pure organic molecules [1, 2]. The inorganic nanomaterials that have been frequently utilized to prepare nanolubricating oils include (1) zero-dimensional spherical or quasi-spherical nanoparticles, such as soft metal nanoparticles, oxide nanoparticles, boron-based nanoparticles, fullerenes, and WS_2_/MoS_2_ hollow nanoparticles [3–12]; (2) one-dimensional nanotubes/nanowires, i.e., carbon nanotubes and MoS_2_ nanotubes/nanowires [13–15]; and (3) two-dimensional (2D) nanoplatelets, such as graphene, MoS_2_ nanosheets, layered metal phosphates, nanoclays, and layered double hydroxides [16–21]. The possible mechanisms that are responsible for the enhanced lubricating performance by applying nanoparticles may vary with the material composition, size, structure, and so on [22–24]. As for the lubricating nanomaterials with different dimensions, 2D layered nanoplatelets are of particular interest due to their anisotropic geometry, high aspect ratio, and efficient lubrication through interlayer sliding and exfoliation [25–28].

Among the 2D lubricating materials used, α-zirconium phosphate (ZrP) and its derivatives are a new but increasingly important class of layered inorganic nanomaterials that have shown excellent friction and anti-wear properties in oil mediums. Recent reports on using ZrP in lithium greases demonstrate that pristine layered ZrP perform much better than MoS_2_, especially under heavy load regime, which is probably due to the stable and rigid 2D molecular frame and strong interlayer bonding of ZrP nanoplatelets [29]. Earlier work in mineral oil show that ZrP and ZrP derivatives exhibit excellent friction behavior at higher load-carrying conditions and anti-wear capacities in liquid oil mediums as compared with traditional lubricating additives, such as MoS_2_ and graphite [30]. It has also been revealed recently that ZrP nanoplatelets are effective in reducing friction in both aqueous and non-aqueous mediums, which is mainly because of the nanoplatelet-induced viscosity modification of the liquid mixtures and the absorption of lubricating molecules on the surfaces of the 2D nanoplatelets [31, 32].

Owing to its defined chemical structure, ease to control the size and aspect ratio, large ion and proton exchange capacity, and high surface and interlayer reactivity for modifications [33–35], ZrP is often regarded as a model 2D nanosystem for studying polymer nanocomposites, drug and biomolecule nanocarriers, lyotropic discotic liquid crystals, and so on [36–43]. Although the utilization of ZrP in nanolubricating oils seems very promising according to the recent research accomplishments, many detailed studies are still lacking before these special 2D layered inorganic nanoplatelets can be applied into practical uses. Such investigations may include the effects of size, thickness, and polydispersity, dispersion states and colloidal stability, surface and interlayer modifications, and so on. ZrP nanoplatelets are hydrophilic; therefore, oil-soluble surfactants are required in order to make them stably dispersed in oil mediums for tribological applications. In a very recent study, organic amines with different alkyl chain lengths have been used to intercalate and thus expand the interlayer spacing of ZrP nanoplatelets in mineral oil for lubricating studies [32]. However, such intercalating molecules would inevitably and non-selectively attach both in between the layers and the outer surfaces of ZrP nanoplatelets. Therefore, it is necessary to develop a specific surface modification method to prepare oil-soluble ZrP nanoplatelets and leave their interlayer structure unattained for further justifications. In such a fashion, the surface and interlayer modifications of ZrP nanoplatelets could be realized separately and the effects on these two factors can thus be studied individually.

In this study, we aim to differentiate the surface and interlayer modifications of ZrP nanoplatelets in order to sort out each effect on the tribological performance in mineral oil. We first attached silane coupling agents with different alkyl chain lengths onto the outer surface of ZrP nanoplatelets to increase their oil solubility and study the effect of surfactant molecule length on their lubricating efficiency in mineral oil. Such surface-modified ZrP nanoplatelets were then intercalated with alkyl amines to further investigate the interlayer modification effects. Through these designed experiments, we have found that surface modification of ZrP nanoplatelets with a long alkyl chain and subsequent intercalation with short amine molecules are the most efficient in terms of reducing friction and wear in mineral oil. Our results demonstrate the feasibility of tuning surface and interlayer functionalities of ZrP nanoplatelets for optimizing their tribological properties in oil mediums, which would be of great benefit in designing practical applications of lubricating oils containing ZrP nanoplatelets.

## Methods

### Synthesis of Pristine ZrP Nanoplatelets

Pristine ZrP nanoplatelets were synthesized using a hydrothermal method developed by Sun et al. [35] In a typical procedure, a sample of 4.0 g ZrOCl_2_·8H_2_O (99.9%, Aladdin) was first mixed with 40.0 ml H_3_PO_4_ (6.0 M) and then sealed into a Teflon-lined pressure vessel. The sample was heated and maintained at 200 °C in an oven for 24 h. After being cooled down to the room temperature, the sample was washed by centrifugation for five times using deionized water to remove excessive H_3_PO_4_. The purified ZrP nanoplatelets were dried at 80 °C in an oven for 24 h and then ground with a mortar and pestle into fine powders before further uses. This sample is identified as pristine ZrP.

### Surface Modification of Pristine ZrP Nanoplatelets

Ten grams of pristine ZrP and 20 g of three alkyl silanes (> 95%, Aladdin), including trimethoxyoctylsilane (C8), dodecyltrimethoxysilane (C12), and hexadecyltrimethoxysilane (C16), were first dissolved by toluene in a 500-mL three-necked flask, respectively. The mixtures were then placed into an oil bath at 100 °C with a constant stirring for 48 h. After the reaction, the solvents were removed by centrifugation and the solid samples were washed by centrifugation for three times using petroleum ether. The surface-modified ZrP nanoplatelets were dried at 70 °C in an oven for 24 h. Finally, the dried ZrP samples were ground with a mortar and pestle into fine powders before further uses. These three surface-modified ZrP nanoplatelets were identified as C8-ZrP, C12-ZrP, and C16-ZrP, respectively.

### Interlay Modification of ZrP Nanoplatelets

Two grams of surface-modified ZrP nanoplatelets (C8-ZrP, C12-ZrP, and C16-ZrP) and primary alkyl amines including 5 g of hexylamine (N6) and 10 g of 1-dodecanamine (N12) were dissolved in 60 mL hexane using a 100-mL glass bottle, respectively. The mixtures were then treated by ultrasonication (40 kHz) for 3 h at room temperature. After ultrasonic treatment, the samples were washed by centrifugation for three times using petroleum ether. The intercalated ZrP nanoplatelets were dried at 70 °C in an oven for 24 h. These six intercalated ZrP samples with different surface modifications were identified as C8-ZrP-N6, C8-ZrP-N12, C12-ZrP-N6, C12-ZrP-N12, C16-ZrP-N6, and C16-ZrP-N12, respectively.

### Preparation of Nanolubricating Oils Containing ZrP Nanoplatelets

The concentration of ZrP nanoplatelets with various modifications in oils was determined to be 0.1 wt% for tribological studies. Master batch oils containing 1.0 wt% of different ZrP samples were first prepared by directly mixing each solid powder with mineral oils under mechanical stirring, followed by ultrasonication for about 20 min to obtain homogeneous oil mixtures. Each stock oil mixture was then diluted to 0.1 wt% using base mineral oil under ultrasonication.

### Characterizations

Crystal structures of all the solid samples were analyzed by their X-ray diffraction (XRD) patterns obtained through a Rigaku X-ray diffractometer system (DMAX-2500, Japan). Scanning electron microscopy (SEM) studies were carried out using a TESCAN Electron Microscope (Vega3, The Czech Republic) operated at 30 kV. Fourier transform infrared spectroscopy (FTIR) was performed using a PerkinElmer Spectrum Two.

**Fig. 1:**
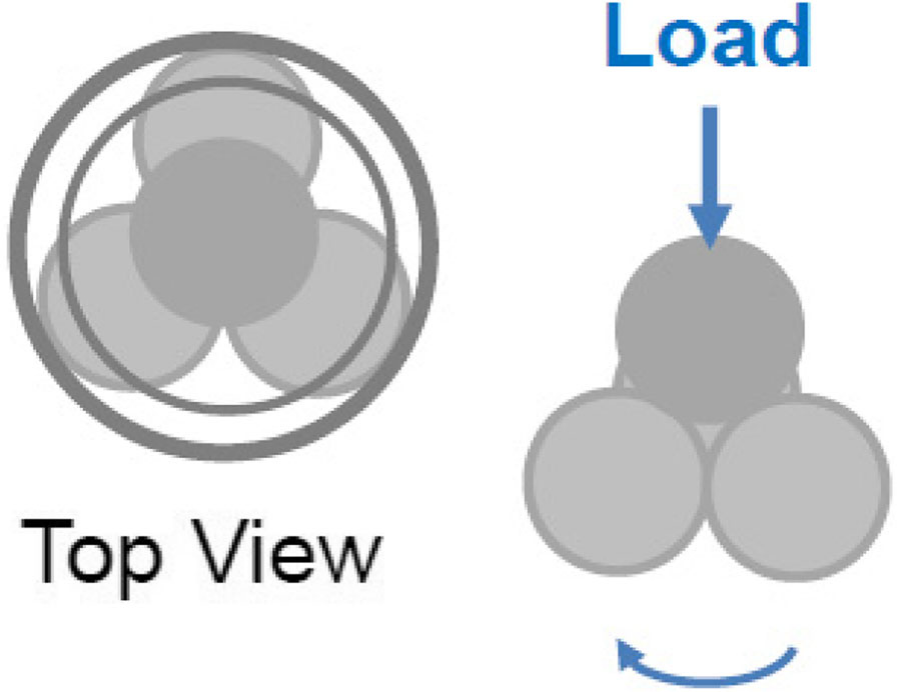
Schematic diagram of the four-ball testing method

Friction and anti-wear properties of nanolubricating oils containing ZrP nanoplatelets with various modifications were tested using a Bruker’s Universal Mechanical Tester (UMT-2, Germany) equipped with a four-ball test setup with ASTM D4172 Standard test method. The testing method is shown in Fig. 1. Before each test, the ball holder was washed with petroleum ether and the metal balls (stainless steel and 12.7 mm in diameter) were cleaned ultrasonically in alcohol. The holder and metal balls were then thoroughly dried. Three metal balls were clamped together in the groove and covered with about 10 mL lubricating oil. The fourth metal ball, referred to as the “top ball,” was then placed on the top of the other three metal balls in the holder. The tester was operated with the top ball held stationary against the other three balls under preset normal loads at room temperature. The coefficients of friction (COFs) for each individual testing were read with time, and the testing duration was 1 h or 3600 s for all the samples. The data were collected at an interval of 100 data point per second. The surface roughness of the metal balls was examined using a Bruker 3D profiler. The average surface roughness of five metal balls is 155.0 ± 14.8 nm (see Additional file 1: Fig. S1). The wear scars on the worn metal balls after testing were examined by a Lecia DM2700 optical microscope. Each lubricating oil sample was measured for five times individually, and the average COF for each sample from these five measurements was calculated.

## Results and Discussion

The individual ZrP layer is covered with hydroxyl groups extending on both sides of the monolayer. In pristine ZrP nanoplatelets, these layers are stacked through relatively strong hydrogen bonding, while their outer surfaces are covered with free hydroxyl groups. The most common method to modify layered ZrP nanoplatelets is to use amine molecules such as alkyl amines or polyether amines [34]. The acid-base reaction between amine groups and hydroxyl groups make these amine molecules not only to attach on the outer surfaces of layered ZrP nanoplatelets but also to be able to intercalate in between ZrP layers. Therefore, in order to modify the surface and interlayer of ZrP nanoplatelets differently, a step-by-step modification method should be developed, and a feasible way to achieve this strategy is to modify and protect the outer surfaces of the pristine ZrP nanoplatelets through covalent bonding first, leaving the interlayer untouched for further intercalation.

**Fig. 2:**
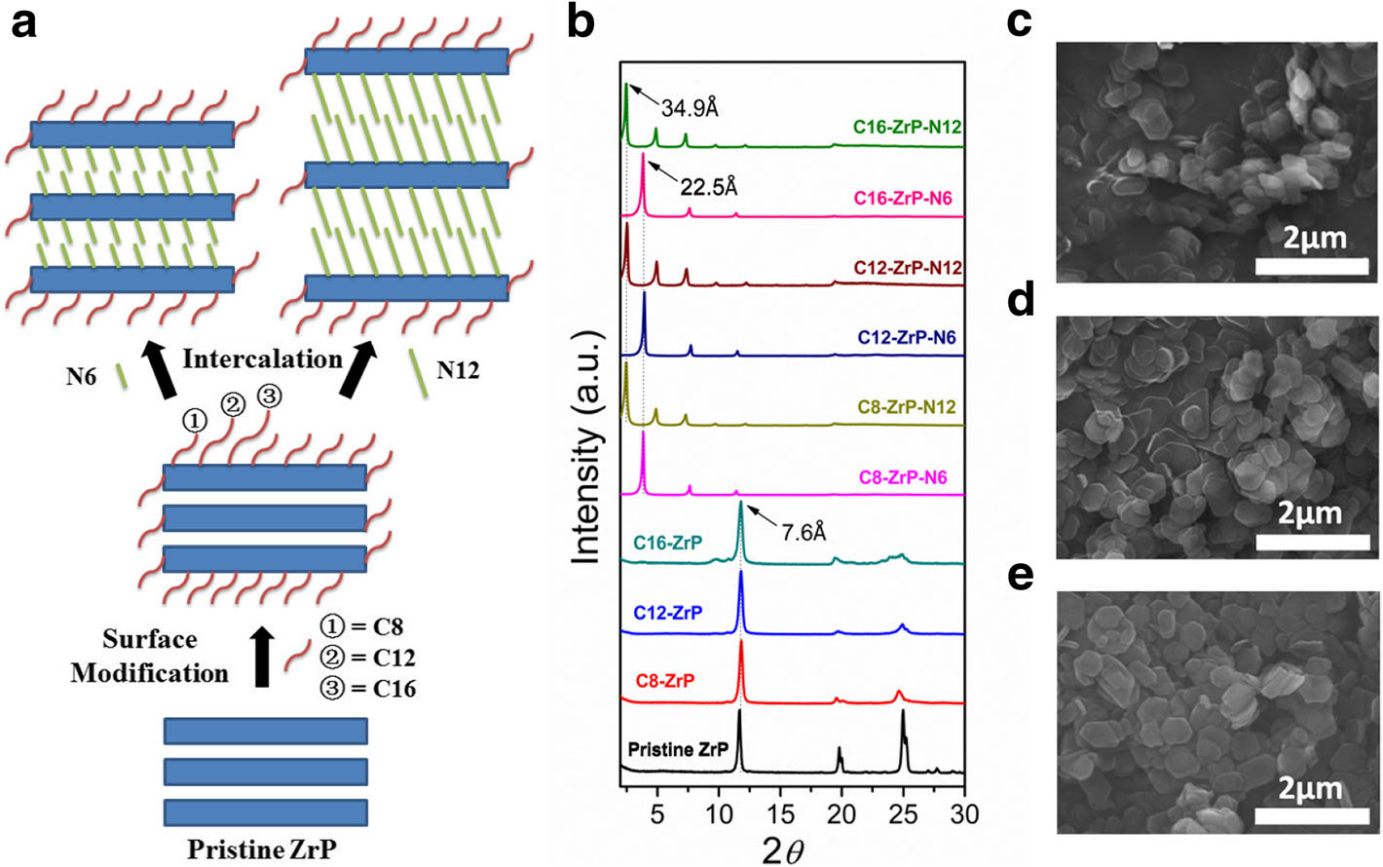
Surface and interlayer modifications of ZrP nanoplatelets: **a** schematic illustration of sample preparations. **b** XRD patterns. SEM images of **c** pristine, **d** surface-modified, and **e** surface-modified and intercalated ZrP nanoplatelets

Figure 2a illustrates our design to achieve different surface and interlayer modifications of ZrP nanoplatelets. We first used a silane coupling method developed in the literature to modify the outer surfaces of pristine ZrP nanoplatelets through covalent bonding [44]. In this step, three alkyl silanes (C8, C12, and C16) were utilized not only to increase the oil solubility of ZrP nanoplatelets but also to investigate the surfactant molecule length effect on the tribological properties of modified ZrP nanoplatelets in oils. FTIR results (see Additional file 1: Figure S2) show the strong characteristic bands associated with the asymmetric and symmetric stretching of the C−H, between 2900 and 3000 cm^−1^, and the appearance of characteristic stretching of the Si–O–P at about 1130 cm^−1^, which demonstrate the successful grafting of silane groups onto the nanoplatelet surfaces [44]. Next, for each silane-modified ZrP nanoplatelets, two different alkyl amines (hexylamine, N6, and 1-dodecanamine, N12) were introduced to intercalate in between layers. In such a fashion, ZrP nanoplatelets with different surface and interlayer modifications can be realized.

**Fig. 3:**
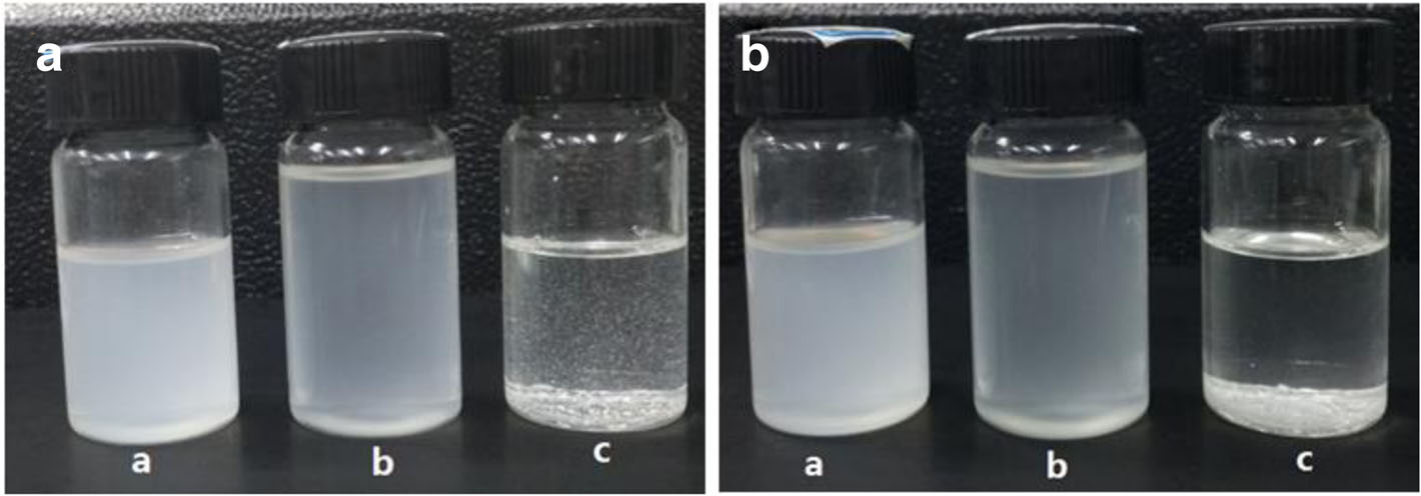
Photographic images of ZrP nanoplatelets in mineral oils **a** right after dispersion and **b** after dispersion for 2 h. Sample a C16-ZrP, sample b C16-ZrP-N6, and sample c pristine ZrP. The concentration of each sample is 0.1 wt%

To validate our strategy, XRD measurements were performed for all the prepared ZrP samples, and the corresponding XRD patterns are shown in Fig. 2b. The samples C8-ZrP, C12-ZrP, and C16-ZrP, representing the silane-modified ZrP nanoplatelets, show the same interlayer spacing of 7.6 Å to pristine ZrP, demonstrating that all the silane molecules used in the current study are unable to intercalate ZrP interlayers and that this first-step modification only occurs on the outer surfaces of ZrP nanoplatelets. This phenomenon is mainly due to the relatively large size of silane molecules that prevents them from entering the interlayers of ZrP nanoplatelets [44]. After introducing alkyl amines, the increasing of interlayer spacing of ZrP nanoplatelets is expected as illustrated in their XRD patterns. The different silane-modified samples that are intercalated with hexylamine (C8-ZrP-N6, C12-ZrP-N6, and C16-ZrP-N6) have the same interlayer spacing of 22.5 Å. When intercalated with 1-dodecanamine, all three samples (C8-ZrP-N12, C12-ZrP-N12, and C16-ZrP-N12) show a larger interlayer spacing of 34.9 Å due to the use of longer alkyl amine molecules. Figure 2c–e shows the representing SEM images of pristine ZrP nanoplatelets, silane-modified ZrP nanoplatelets, and silane-modified ZrP nanoplatelets with amine intercalations, respectively. All these three types of ZrP samples have a platelet structure with a similar diameter of around 600–800 nm, indicating that the surface and interlayer modifications do not affect the plate-like morphology and the diameter of ZrP samples. The above characterization results also suggest that such prepared samples would provide an ideal model for systematically investigating the surface and interlayer effects on the tribological performance of ZrP nanoplatelets in oils. The representative dispersion stability of various ZrP samples in mineral oils is shown in Fig. 3. The ZrP nanoplatelets with surface and interlayer modifications can be homogeneously and stably dispersed in mineral oils. However, the pristine ZrP nanoplatelets without any functionalization are insoluble in oil and sediment quickly onto the bottom. Therefore, the oil samples containing pristine ZrP nanoplatelets are not suitable for nanolubricating oil applications and thus were not tested in the current study.

**Fig. 4:**
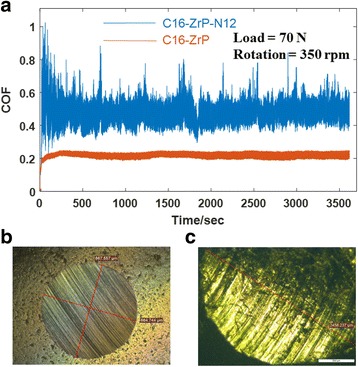
**a** Friction coefficients of surface-modified ZrP nanoplatelets without and with intercalations (C16-ZrP and C16-ZrP-N12) in mineral oils under a load of 80 N and rotation speed of 350 rpm. Optical microscopic images of the wear scar images for **b** C16-ZrP and **c** C16-ZrP-N12 in mineral oils after testing

Tribological measurements of mineral oils containing various types of ZrP nanoplatelets with the concentration of 0.1 wt% were performed using a four-ball module under a load of 70 N and rotation speed of 350 rpm in 1 h, and the wear scars after the four-ball testing were examined by optical microscopic imaging. Figure 4 shows the selected raw data (C16-ZrP and C16-ZrP-N12 in mineral oils) from our friction and wear testing. The COFs were measured as a function of time, and the fluctuation of the COF data in each measurement is an indication of lubricating stability for the tested oil sample. In the case of the COFs for C16-ZrP and C16-ZrP-N12 in mineral oils, as shown in Fig. 4a, the silane-modified ZrP nanoplatelets after intercalated with 1-dodecanamine exhibit a much higher COF (~ 0.50 vs. ~ 0.20) with a much larger range of COF data fluctuation during the whole testing period of 1 h as compared to the same surface-modified ZrP nanoplatelets but without any alkyl amine intercalation. Moreover, C16-ZrP in mineral oil produces a rather smooth and circular wear scar with a diameter of around 600 μm after the four-ball testing as observed in Fig. 3b, while the wear damage from C16-ZrP-N12 in mineral oil shown in Fig. 3c is very rough and elliptical in shape with a long diameter of around 2400 μm. By considering both the COF and wear scar imaging results shown in Fig. 4, it is suggested that a large increase in interlay spacing of ZrP nanoplatelets, i.e., from the pristine 7.6 to 34.9 Å by 1-dodecanamine intercalation, would cause a significant drop in lubricating efficiency for the nanolubricating oils.

**Fig. 5:**
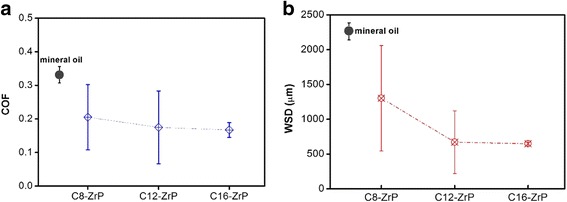
**a** Friction coefficients and **b** wear scar diameters of surface-modified ZrP nanoplatelets

Tribological performances including both COF and WSD results for all the nanolubricating oils containing surface-modified ZrP nanoplatelets are illustrated in Fig. 5a, b, respectively. The average COF and average WSD for the base mineral oil are also shown in the corresponding figure for the purpose of comparison. The base mineral oil shows an average COF of about 0.33 and an average WSD of about 2300 μm. All the nanolubricating oil samples containing saline-modified ZrP nanoplatelets of various alkyl chain lengths (C9-ZrP, C12-ZrP, and C16-ZrP) exhibit lower average COFs and smaller WSDs than the base mineral oil, suggesting that better tribological performance can be achieved by adding surface-modified ZrP nanoplatelets without any intercalation in mineral oil.

The average COF and average WSD of nanolubricating oils decrease as the increase of the alkyl chain length on the outer surface of the silane-modified ZrP nanoplatelets as shown in Fig. 5a, b, respectively. The C8-ZrP lubricating oil sample has an average COF of about 0.20, which is ~ 40% lower than the base mineral oil sample. The nanolubricating oils containing C12-ZrP and C16-ZrP show average COFs of about 0.18 and 0.17, respectively, which are slightly lower than the nanolubricating oil with C8-ZrP. As for the wear testing results, the nanolubricating oils with C8-ZrP, C12-ZrP, and C16-ZrP show average WSDs of ~ 1300, ~ 700, and ~ 600 μm, respectively, which are about 43, 70, and 74% smaller than the base mineral oil, respectively. The above tribological results may be because of the fact that a longer alkyl chain on the surface of ZrP nanoplatelets would lead to a better dispersion and thus a better friction and anti-wear behavior for the prepared nanolubricating oils. Moreover, it is interesting to note that the error variations for both COF and WSD of the nanolubricating oil containing C16-ZrP are much smaller than those of the oils with C8-ZrP and C12-ZrP, and even smaller than the pure mineral oil, which might be also due to the better dispersion of surface-modified ZrP nanoplatelets with longer alkyl chains. The tribological performance of nanolubricating oils is highly dependent on nanoparticle dispersions. The presence of large aggregates in the poor nanoparticle-oil dispersions may cause relatively large-scale inhomogeneities in the lubricating mediums, leading to an unstable rheological behavior and a poor tribological performance upon friction. When the nanoplatelets are well-dispersed in oils, however, the homogenous oil dispersions could provide a smooth lubrication between the friction surfaces where the dispersed nanoplatelets would function well as lubrication-enhancing nano-agents and a superior and stable tribological performance can thus be achieved.

**Fig. 6:**
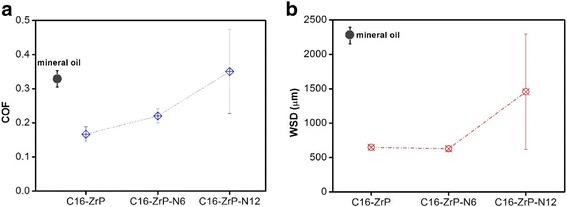
**a** Friction coefficients and **b** wear scar diameters of surface-modified ZrP nanoplatelets without and with intercalations

The silane-modified ZrP nanoplatelets with the longest alkyl chain (C16-ZrP), which show the best tribological performance in mineral oils in all the surface-modified samples prepared, were intercalated with two alkyl amines, hexylamine (N6) and 1-dodecanamine (N12), to investigate the interlayer modification effect on the friction and anti-wear properties of nanolubricating oils. Figure 6a, b shows the COFs and WSDs of nanolubricating oils containing C16-ZrP, C16-ZrP-N6, and C16-ZrP-N12 as compared with the pure mineral oil, respectively. The average COFs of these nanolubricating oils increase as the increase of the interlayer distances by the alkyl amine intercalation. The average COF of the nanolubricating oil with C16-ZrP-N6 is about 0.21, which is higher than that of the C16-ZrP oil sample (~ 0.17), but is still ~ 36% lower than that of mineral oil (~ 0.33). However, the nanolubricating oil with C16-ZrP-N12 exhibits a much higher average COF of about 0.35, even higher than the pure mineral oil with an average COF of about 0.33. As for the observed wear damages, the average WSD for the nanolubricating oil with C16-ZrP-N6 is about 550 μm, even a little bit smaller than that of the C16-ZrP oil sample (~ 600 μm). The nanolubricating oil containing C16-ZrP-N12 with a larger interlay spacing, however, exhibits a much larger average WSD (~ 1400 μm) than the C16-ZrP- and C16-ZrP-N6-containing oil samples.

The above COF and WSD results shown in Fig. 6 suggest that a small increase in the interlayer spacing for the surface-modified ZrP nanoplatelets by the alkyl amine intercalation, i.e., from the original layer spacing of 7.6 to 22.5 Å by the hexylamine intercalation, would not cause a significant change in the friction and anti-wear properties of nanolubricating oils under the current testing conditions. On the contrary, when the silane-modified ZrP nanoplatelets are intercalated by amine molecules with a longer alkyl chain, i.e., 1-dodecanamine with an interlayer spacing of 34.9 Å, a drastic reduction in the tribological performance for such prepared nanolubricating oil can be observed, which somehow is even worse than the pure mineral oil in terms of the friction coefficient. Moreover, as seen in Fig. 6a, b, the error variations of both COF and WSD for the C16-ZrP-N12-containing oil sample are significantly larger than those of both C16-ZrP and C16-ZrP-N6 oils, indicating that the large increase in the interlayer spacing of the surface-modified ZrP nanoplatelets by the intercalation of 1-dodecanamine causes an extremely unstable tribological performance for the corresponding nanolubricating oil. This phenomenon may be explained by the large increase in thickness of ZrP nanoplatelets and the structure instability upon intercalation by 1-dodecanamine.

**Fig. 7:**
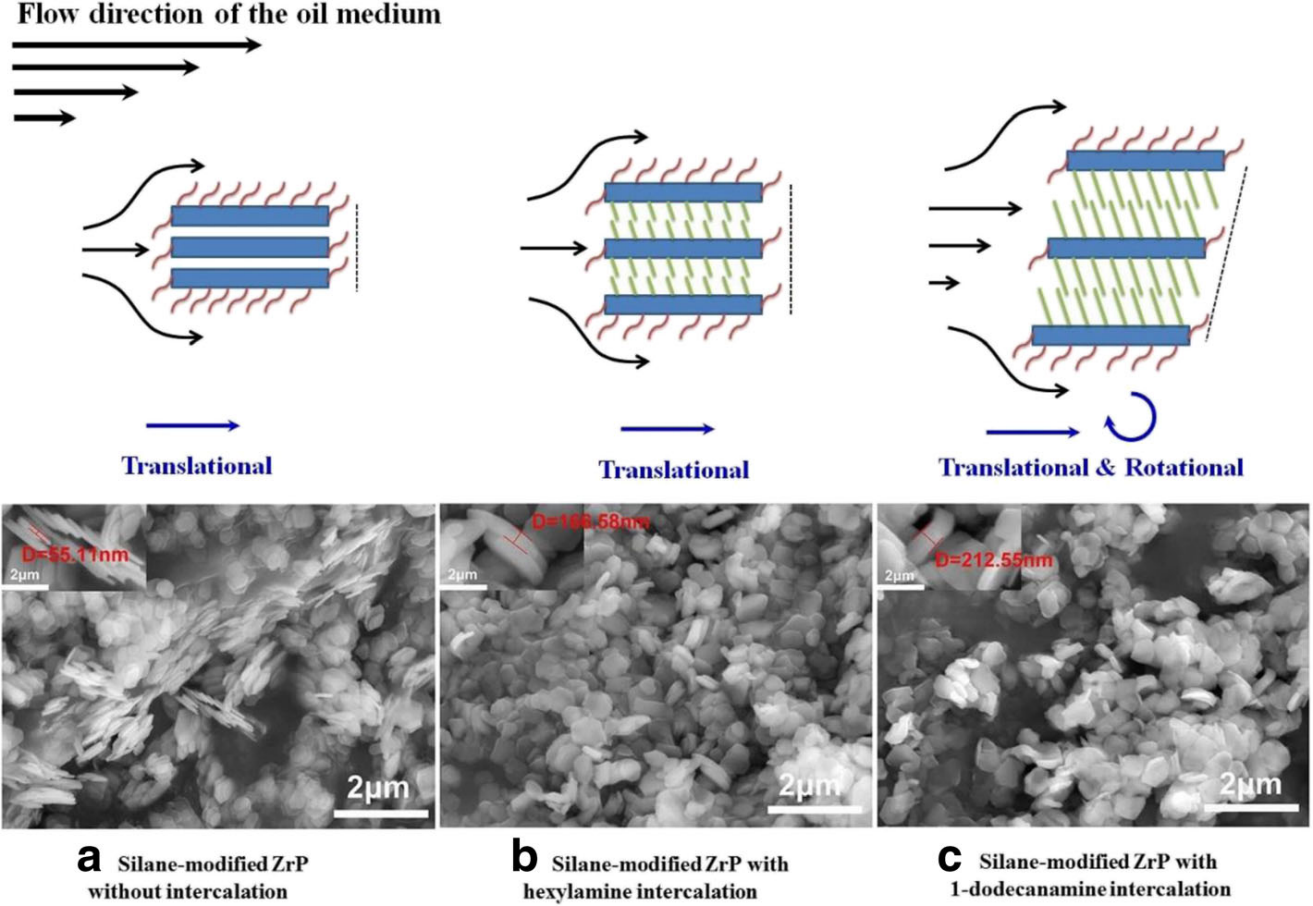
Proposed rheological behaviors of surface-modified ZrP nanoplatelets with and without intercalations in oils. The layered nanoplatelets shown in the cartoon are not drawn to scale. The bottom is the corresponding SEM images of surface-modified ZrP nanoplatelets with and without intercalations. **a** Silane-modified ZrP nanoplatelets without intercalation (thickness is ~ 55 nm). **b** Silane-modified ZrP nanoplatelets with hexylamine intercalation (thickness is ~ 160 nm). **c** Silane-modified ZrP nanoplatelets with 1-dedecanamine intercalation (thickness is ~ 210 nm)

The ZrP nanoplatelets synthesized in this study have an average diameter of 600–800 nm as observed in the SEM images in Fig. 2. The thickness of the pristine and surface-modified ZrP nanoplatelets based on both our SEM images and the literature report is about 70 nm, resulting in a diameter-to-thickness/aspect ratio of ~ 10, neglecting the slight increase in thickness by the silane modifications. The intercalations by hexylamine and 1-dodecanamine lead to around twofold and fourfold increases in the thickness of ZrP nanoplatelets, respectively, and thus cause the decrease in the nanoplatelet aspect ratios. It has been found recently that the intercalations of pristine ZrP nanoplatelets with small amine molecules such as ethyleneamine, propylamine, and butylamine in oils help increase the lubricating performance, which results from the improved rheological properties of nanolubricating oils [32]. In our study, the nanolubricating oils containing C16-ZrP and C16-ZrP-N6 also exhibit better tribological performance than the pure mineral oil, which agrees well with the above literature finding. However, the observed drastic decrease in the lubricating behavior by further increasing the interlayer spacing with 1-dodecanamine intercalation may be attributed to the size and dimension changes of ZrP nanoplatelets due to the increase of their thickness and the reduction of their aspect ratio. Furthermore, when the aspect ratio of the nanoplatelets in oils is large as in the case of our C16-ZrP and C16-ZrP-N6 and the directly intercalated ZrP nanoplatelets with small amine molecules reported in the literature [32], the movement of nanolubricating oils during the friction process would cause the alignment and the translational motion along the direction of the oil flow for most of the dispersed nanoplatelets, which helps improve the rheological properties of the oil medium. However, when the aspect ratio of the nanoplatelets is largely decreased, the shear force induced by the motion of the oil medium would inevitably cause the rotations of such large in size but small in aspect ratio nanoplatelets, thus resulting in reduced rheological behavior and poor tribological performance. In addition, when ZrP nanoplatelets are intercalated by 1-dodecanamine, the large interlayer spacing dramatically reduces the interactions between individual layers in each intercalated nanoplatelets. Therefore, the shear stress applied on the dispersed C16-ZrP-N12 might also cause a large deformation of the intercalated nanoplatelets and, to some extent, affect their structure integrity, thus leading to the worse tribological performance as compared to the ZrP nanoplatelets with smaller interlayer distances. The proposed mechanism to explain the above phenomenon is illustrated in Fig. 7.

**Fig. 8:**
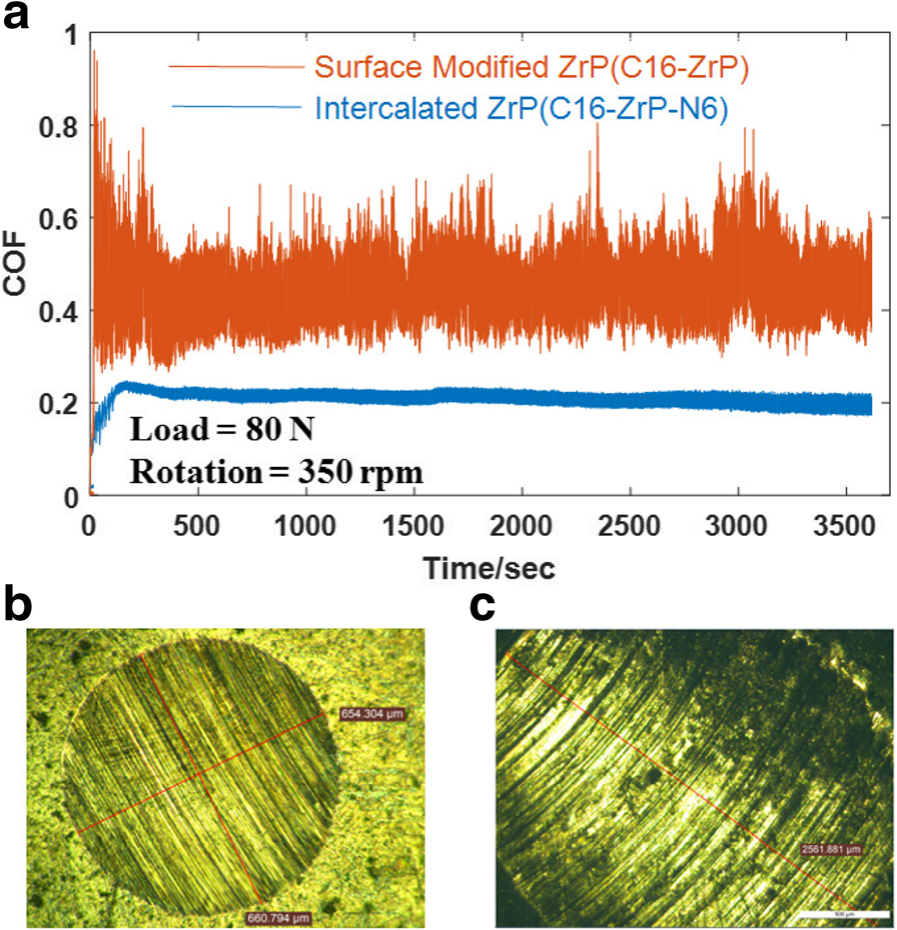
**a** Friction coefficients of the nanolubricating oils containing C16-ZrP and C16-ZrP-N6 under a load of 80 N and rotation speed of 350 rpm. Optical microscopic images of the wear scar images for **b** C16-ZrP-N6 and **c** C16-ZrP in mineral oils after testing

**Fig. 9:**
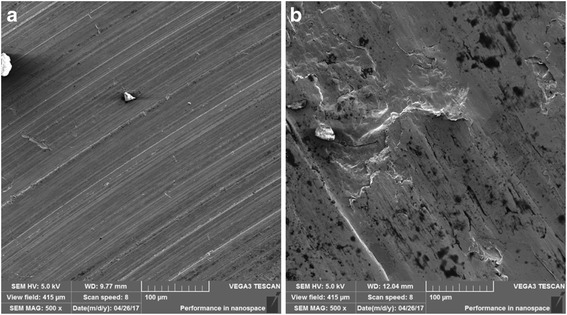
SEM images of the wear scars for **a** C16-ZrP-N6 and **b** C16-ZrP in mineral oils after testing under a load of 80 N and rotation speed of 350 rpm

The tribological results from our four-ball testing under a load of 70 N and rotation speed of 350 rpm, as summarized in Figs. 5 and 6, suggest that C16-ZrP and C16-ZrP-N6 in mineral oils perform the best in terms of their COFs and WSDs under such testing condition. These two nanolubricating oil samples were next tested under an increased load of 80 N and the same rotation speed of 350 rpm to examine their tribological performance under a higher load condition, and the corresponding COFs and wear scar images are shown in Fig. 8. The COF of nanolubricating oil containing C16-ZrP for this individual test is about 0.45 with a very large range of data fluctuation as shown in Fig. 8a, indicating a poor and unstable lubricating behavior under an increased load of 80 N as compared to the relatively low and stable COF profile (~ 0.20 for the individual test shown in Fig. 4a and ~ 0.17 for the average COF) obtained under a load of 70 N. On the contrary, under this increased load condition, the COF profile of the nanolubricating oil with C16-ZrP-N6 is smooth with rather small data fluctuations and its COF is about 0.20, which is very close to the average COF (~ 0.21) of the same sample under a load of 70 N. The wear damage under the load of 80 N for the C16-ZrP-N6 oil sample is about 650 μm in diameter as shown in Fig. 8b, which is a reasonable increase as compared to the WSD of ~ 550 μm for the same sample under the load of 70 N. However, for the C16-ZrP oil sample tested under the load of 80 N, the wear damage, as shown in Fig. 8c, becomes very large and elliptical in shape with a long diameter of around 2600 μm, a dramatic increase as compared to the same sample tested under the load of 70 N (round wear scar of ~ 600 μm in diameter). The corresponding SEM images of the above two samples are illustrated in Fig. 9. Similar to the observation in Fig. 8b, c, the wear surface of the C16-ZrP-N6 oil sample is much smoother than that of the C16-ZrP oil sample. The above results suggest that a small increase in the interlayer spacing with relatively small amine molecules, i.e., hexylamine, would lead to a better tribological performance of the intercalated ZrP than the nanoplatelets without intercalation in mineral oil. The mechanism that is responsible for the above phenomenon could be due to the balanced interlayer interactions in the layered ZrP nanoplatelets introduced by relatively small amine molecules. The pristine layered crystal structure of ZrP nanoplatelets is rather rigid and brittle, while the hexylamine-intercalated ZrP nanoplatelets should be tougher and more elastic, which makes them more stable and durable under a relatively heavy load, thus leading to a better tribological performance for such layered nanoplatelets in oils. Meanwhile, the elemental analysis on the above two worn surfaces (Additional file 1: Figs. S3–S5) did not have any remaining ZrP nanoplatelets, indicating that the modified ZrP nanoplatelets in the current study may enhance the lubricating efficiency by sliding between the metal friction surfaces, rather than bonding on each metal surface. The detailed mechanisms may be explored by studying individual nanoplatelets of various modifications through micro/nano-mechanical measurements and are under our further investigations. Nevertheless, the large increase in the interlayer spacing, i.e., by 1-dodecanamine intercalation, would certainly cause a poor tribological performance of ZrP nanoplatelets in mineral oil.

## Conclusions

In summary, we have investigated the effects of surface and interlayer modifications on the tribological properties of layered ZrP nanoplatelets in mineral oil. Instead of directly using alkyl amines to intercalate and disperse ZrP nanoplatelets in oils, silane coupling agents with C8-, C12-, and C16-alkyl chains were first utilized to modify the outer surfaces of the pristine ZrP without any intercalations to study the surface modification effect. Such surface-modified ZrP nanoplatelets were further intercalated by hexylamine and 1-dodecanamine to investigate the interlayer modification effect. The standard four-ball tribological measurements on the friction coefficients and wear damages of nanolubricating oils containing various modified ZrP nanoplatelets illustrate that a longer alkyl chain on the outer surfaces will result in a better tribological performance and a further intercalation with 1-dodecanamine will cause a significant decrease in the tribological performance. When the surface-modified ZrP nanoplatelets are intercalated with hexylamine, the tribological behavior of the nanolubricating oil is similar to the one without any intercalation under a load of 70 N. However, when the testing load is increased to 80 N, the surface-modified ZrP nanoplatelets with hexylamine intercalation show much better tribological properties than the ones without any intercalation in mineral oil. Our findings demonstrate the importance of tuning surface and interlayer modifications of 2D-layered nanolubricating additives for better tribological performance and are of great significance in designing high-performance nanolubricating oils for practical uses.

## Additional file

Additional file 1: Figure S1A–E. Surface roughness of five metal balls examined by a 3D profiler. The average surface roughness is 155.0 ± 14.8 nm. **Figure S2.** FTIR of various surface modified-ZrP samples. The strong characteristic bands associated with the asymmetric and symmetric stretching of the C−H, between 2900 and 3000cm^−1^, and bending at ca. 1450 cm^−1^ are an indication of the attachments of alkyl chains from various silanes on ZrP nanoplatelets. **Figure S3.** SEM and EDS results for the original metal surface before testing. **Figure S4.** SEM and EDS results for the worn metal surface after testing with the C16-ZrP-N6 oil sample. **Figure S5.** SEM and EDS results for the worn metal surface after testing with the C16-ZrP oil sample. (DOCX 3672 kb)
