# Supplementary material for: Tuning Tribological Performance of Layered Zirconium Phosphate Nanoplatelets in Oil by Surface and Interlayer Modifications
Source: Nanoscale Res Lett. 2017 Sep 20;12:542. doi: 10.1186/s11671-017-2315-2 (PMC5607051; doi:10.1186/s11671-017-2315-2)
Supplement: Additional file 1: Figure S1A–E. — Surface roughness of five metal balls examined by a 3D profiler. The average surface roughness is 155.0 ± 14.8 nm. Figure S2. FTIR of various surface modified-ZrP samples. The strong characteristic bands associated with the asymmetric and symmetric stretching of the C−H, between 2900 and 3000cm−1, and bending at ca. 1450 cm−1 are an indication of the attachments of alkyl chains from various silanes on ZrP nanoplatelets. Figure S3. SEM and EDS results for the original metal surface before testing. Figure S4. SEM and EDS results for the worn metal surface after testing with the C16-ZrP-N6 oil sample. Figure S5. SEM and EDS results for the worn metal surface after testing with the C16-ZrP oil sample. (DOCX 3672 kb) [file 11671_2017_2315_MOESM1_ESM.docx]

**Supporting information**

**Tuning Tribological Performance of Layered Zirconium Phosphate Nanoplatelets in Oil by Surface and Interlayer Modifications**

Xiao Han, Huaisong Yong, and Dazhi Sun*

Department of Materials Science and Engineering and Shenzhen Key Laboratory of Nanoimprint Technology, Southern University of Science and Technology, Shenzhen 518055, China

*E-mail: [sundz@sustc.edu.cn](mailto:sundz@sustc.edu.cn)


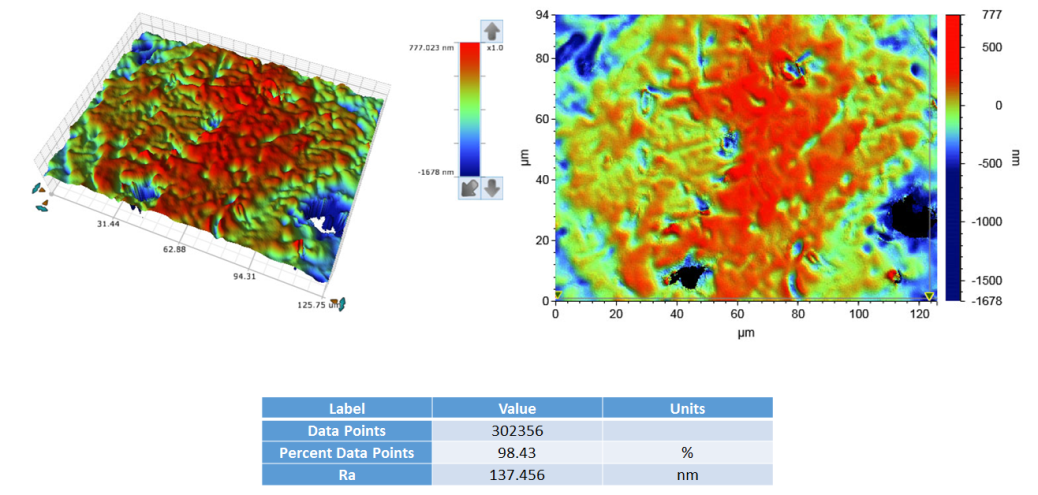


A


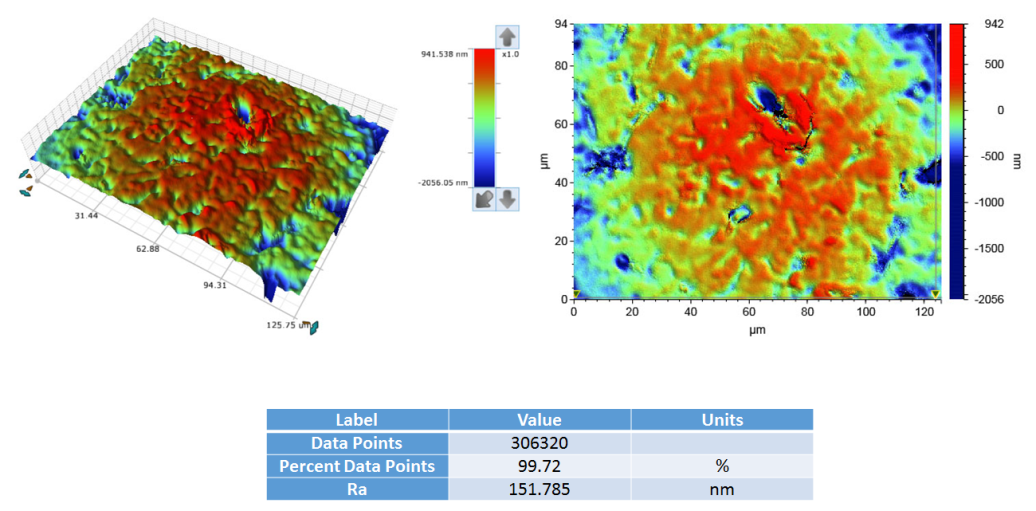


B


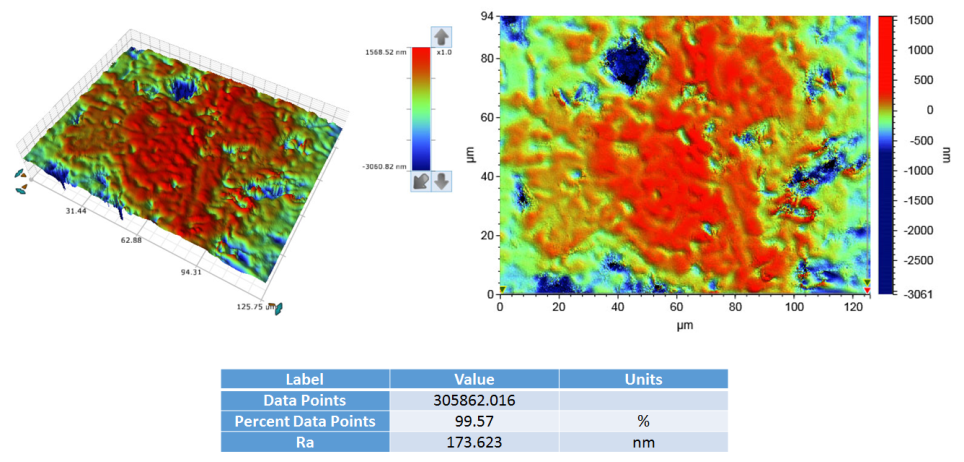


C


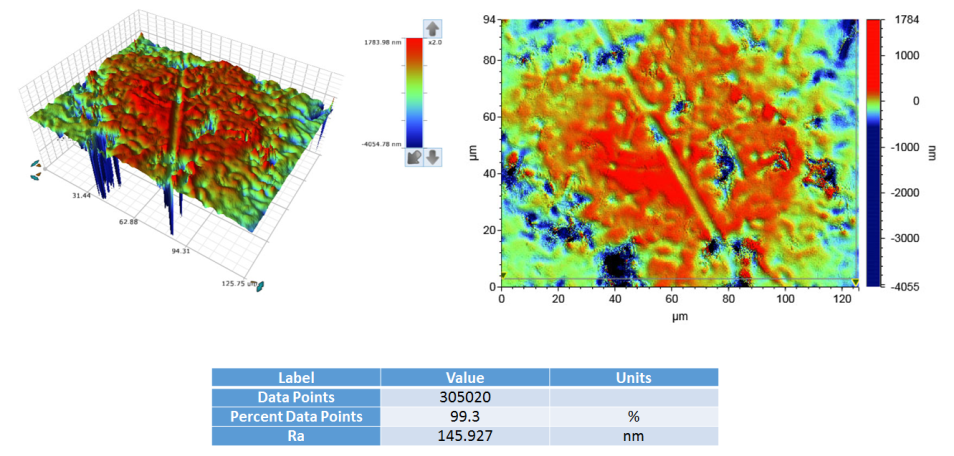


D


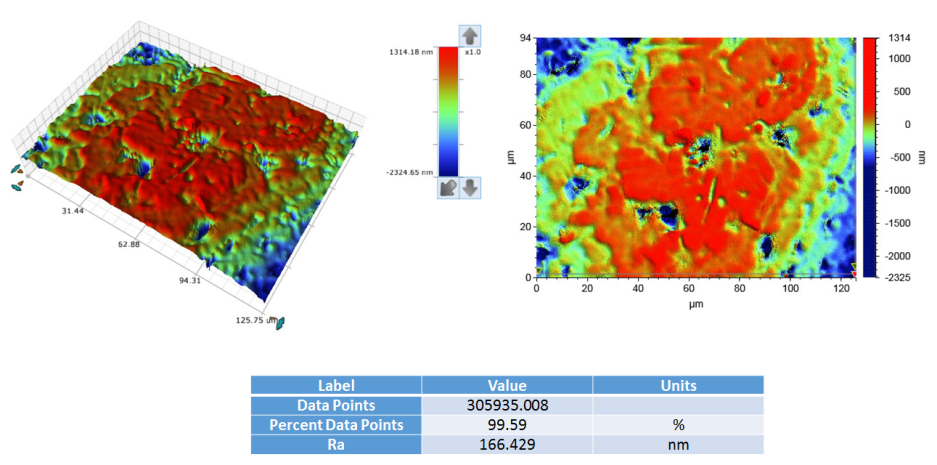


E

**Figure S1A-E.** Surface roughness of 5 metal balls examined by a 3D profiler. The average surface roughness is 155.0 ± 14.8 nm.

**Figure S2.** FTIR of various surface modified-ZrP samples. The strong characteristic bands associated with the asymmetric and symmetric stretching of the C−H, between 2900 and 3000cm^−1^ and bending at ca. 1450 cm^−1^ are an indication of the attachments of alkyl chains from various silanes on ZrP nanoplatelets.

**Surface Composition Analysis**. The elemental analysis on the damaged and undamaged surfaces was performed using an Energy Dispersive Spectrometer (EDS, Bruker, XFLASH® 6|30 detector with area scanning) under a Scanning Electron Microscope (TESCAN, EGA 3 LMH). The EDS results are shown in figure S2-S4. No Zr was found on the worn surfaces after testing, indicating that ZrP nanoplatelets could not attach tightly onto the friction surfaces.


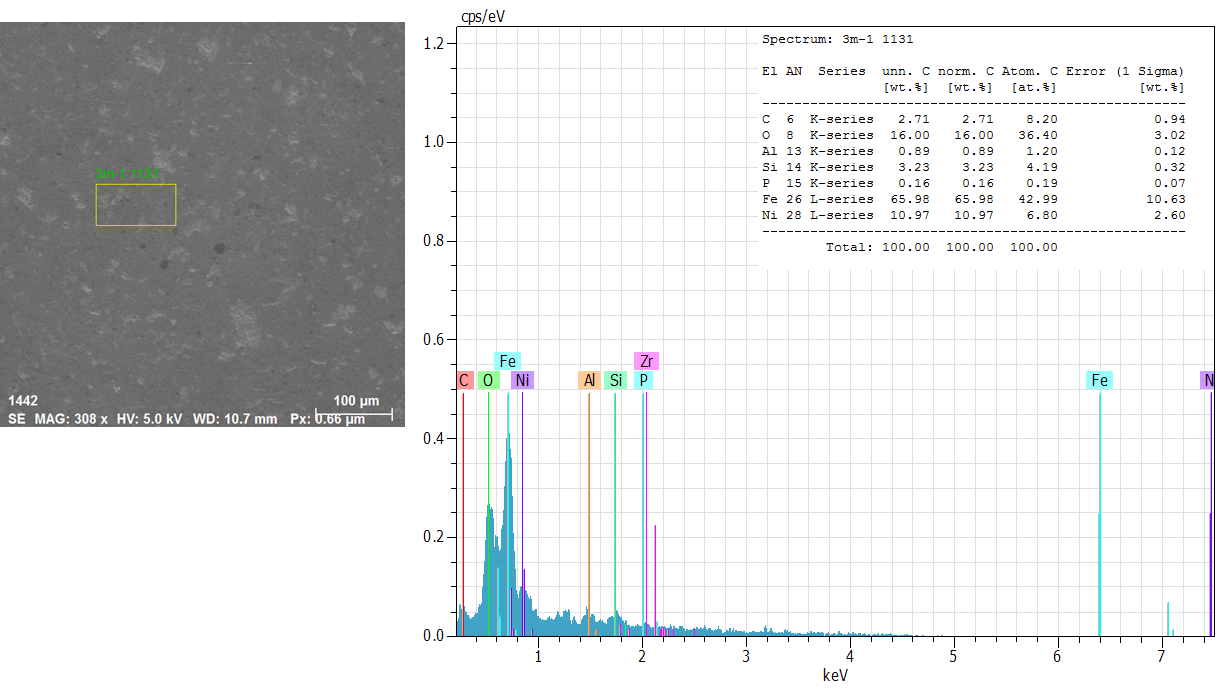


**Figure S3.** SEM and EDS results for the original metal surface before testing.


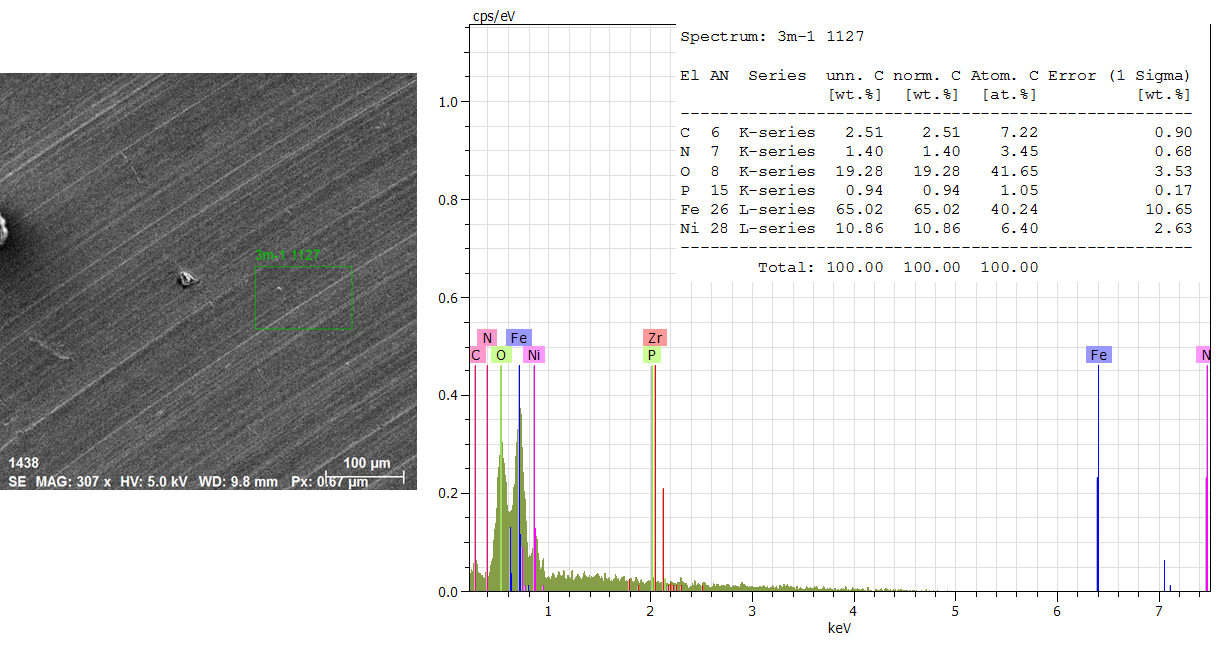


**Figure S4.** SEM and EDS results for the worn metal surface after testing with the C16-ZrP-N6 oil sample.


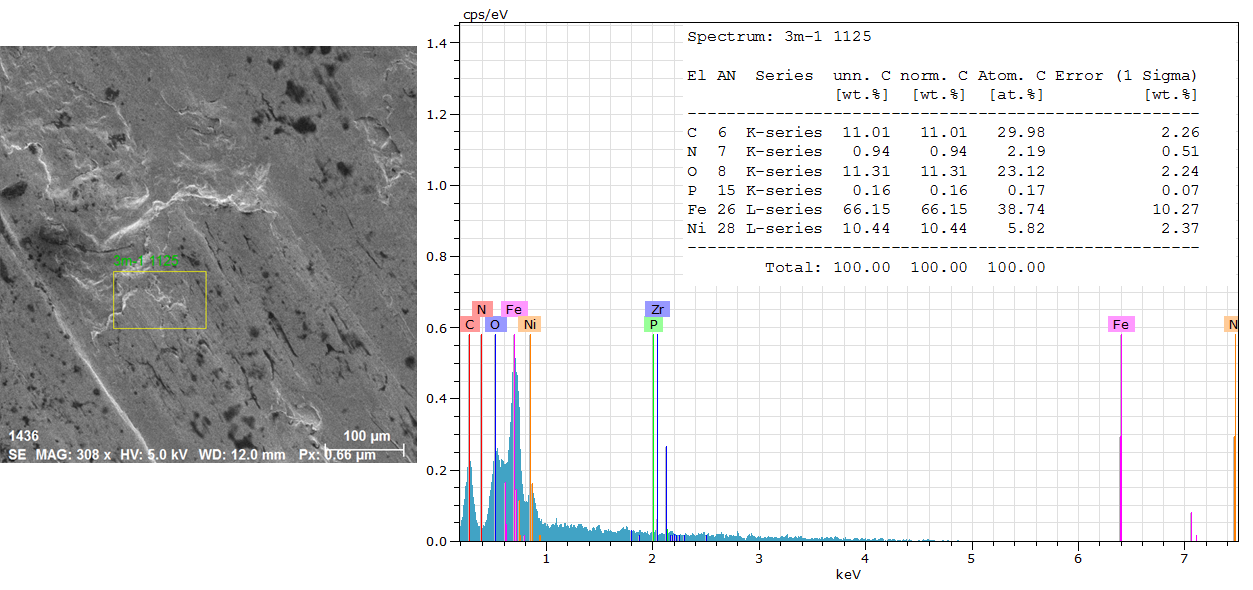


**Figure S5.** SEM and EDS results for the worn metal surface after testing with the C16-ZrP oil sample.
